# Estimating the cost to rural ambulating HIV/AIDS patients on Highly Active Antiretroviral Therapy (HAART) in rural Ghana: a pilot study

**Published:** 2012-06-04

**Authors:** Stephen Apanga, Damien Punguyire, George Adjei

**Affiliations:** 1 School of Public Health University of Ghana, Ghana; 2 Kintampo Health Research Centre Kintampo, Ghana; 3 Kintampo Municipal Hospital Kintampo, Ghana

**Keywords:** HIV, AIDS, Highly Active Antiretroviral Therapy, antiretroviral drugs, indirect cost, Direct cost, Kintampo, rural ambulating

## Abstract

**Background:**

Subsidized antiretroviral therapy programs obviously lowers the cost of antiretroviral drugs but other major costs are still incurred, which makes the overall cost of accessing and consuming antiretroviral treatment very high and sometimes catastrophic. The objective of this study was to estimate the total cost to rural ambulating HIV/AIDS patients on highly active antiretroviral therapy in a rural area of the middle belt of Ghana.

**Methods:**

This was a convenient cross-sectional study of people diagnosed with HIV/AIDS receiving outpatient care and carried out from September to October 2009 involving 80 HIV/AIDS patients on HAART. Data was derived from patients’ medical records on health care utilization and a completed pre tested questionnaire used to obtain the cost of transportation and estimates of individual earned income from which the labor productivity loses (opportunity cost) for days not worked as a result of attending the antiretroviral clinic were derived.

**Results:**

The median total, indirect and direct annual costs to rural ambulating HIV/AIDS patients on HAART were estimated to be $US71.18 (115.16 Ghana cedis), $US2.740 (3.92 Ghana cedis) and $US53.04 (75.00 Ghana cedis) respectively.

**Conclusion:**

Although the cost of antiretroviral drugs has been subsidized by government from $360 to $41.38 per annum, HIV/AIDS patients on HAART spend double of this subsidized amount out of their pocket seeking health care. We recommend that agencies associated with HIV/AIDS activities, supplements government's effort by helping to get antiretroviral closer to the door step of patients so as to reduce this huge financial burden which constitutes more than 100% of their median annual earned income.

## Background

Annual expenditures on HIV/AIDS interventions in resource-poor countries is estimated to have increased about 30-fold over the last decade, to an estimated $US 10 billion in the 2007 [1]. Antiretroviral therapy (ART) has been proven to be extremely effective in resource-poor settings increasing survival for patients with AIDS from 30% to 90% in one year in most cases [2–6]. However, the only few studies that have reported data on the costs of providing ART in resource-poor settings derived from actual patient experience come from South Africa, Thailand, and Mexico [7–9]. Due to advances in therapy since the mid-90s, several reports have documented a shift in HIV/AIDS health service utilization from inpatient to outpatient care in developed countries since the introduction of Highly Active Antiretroviral Therapy (HAART) [10–12].

While providing ARV drugs free of charge has been proven to be an important step in HIV/AIDS care, the costs of other components of care constitute important financial barriers that may exclude patients from accessing appropriate care [13]. Subsidized ART program obviously lowers the cost of ARV drugs but other major costs are still incurred, which may make the overall cost of accessing and consuming ART treatment very high and sometimes catastrophic in most instances [14]. Most economic assessments carried out in this area deal with the determination of direct costs (prevention, diagnosis and treatment of HIV disease). Economic assessments incorporating the costs associated with productivity losses (indirect costs) are uncommon in the health economics literature on HIV/AIDS [10–12]. Very few studies have been conducted in this area in the least developed countries [15–17] with none of these studies collecting patient-level data [18]. In addition, much is hardly known about household costs not covered by health insurances (e.g., transport to the provider and special diet amongst others). Although there are more studies on indirect costs, these studies are difficult to compare due to methodological differences [19, 20]. Average indirect costs increase as HIV-infected individuals′ illness progresses and whether one takes a public-sector or societal perspective, indirect costs add a considerable amount to the cost of delivering health care to HIV-infected individuals. Both direct and indirect costs, when obtainable, should be used to assess the economic consequences of HIV infection and treatment interventions as they present a better picture of the situation [21].

In Ghana, monthly cost of ARV per patient was US$30 though patients were made to pay US$ 5. Total patient expenditure on ART could rise to US$ 55 depending on how far the patients had to travel to get to the nearest ART center and how long they had to wait at the center [22].

The government of Ghana through the national AIDS control program has made antiretroviral drugs free to those who need therapy including laboratory investigations. However, those on the drugs will have to bear some cost themselves which include paying a token monthly, paying for other medical conditions including opportunistic infections and other indirect cost such as transportation cost and waiting time amongst others. Till date very few studies have been done to estimate the cost rural or poor people on HAART have to bear in Ghana which could have untold effects on their lives. The aim of this study is to estimate the total cost to rural ambulating HIV/AIDS patients on HAART which is expected to help serve as a guide for agencies involved in HIV/AIDS activities in the middle belt of Ghana which has one of the highest prevalent rates in Ghana.

## Methods

### Settings, population and study design

This was a cross-sectional retrospective study involving people diagnosed with HIV/AIDS and receiving HAART on outpatient basis at the antiretroviral clinic in the Kintampo municipal hospital in the center of Ghana. This clinic attends to about 400 diagnosed HIV/AIDS people from more than four districts with over 170 of them on HAART as at end of September 2009 [24].

### Subjects and variables of interest

80 HIV/AIDS adult (≥18 years old) clients on HAART attending the antiretroviral clinic for at least 1 year between January 2008 and September 2009 were conveniently sampled from the beginning of September to the end of October 2009. Data on cost was obtained from patients medical records and a completed pre tested questionnaire after signing or thumb printing a confidential informed consent form in the presence of a witness (an independent health worker at the antiretroviral clinic).

### Direct costs

Direct costs to patients were derived from medical records on health care utilization in terms of scheduled monthly visits (payment of monthly fees) and unscheduled visits for both HAART and treatment for opportunistic infections. For the non insured clients, unit cost of drugs prescribed was based on the national health insurance scheme medicines list of October 2008 [25] which the Kintampo Municipal Hospital uses. For the insured clients, annual premiums are paid which caters for both service and drugs. The cost of service for both the insured and the uninsured clients in this study population however remains free.

### Indirect costs

The questionnaire was used to obtain estimates of individual earned income from which the labor productivity loses (opportunity cost) for days not worked as a result of attending the antiretroviral clinic were derived. Unit costs for calculating indirect costs for the unemployed were based on prevailing local market prices whiles that for the formally employed were based on the government of Ghana's fare wages salary structure for the year 2009. The survey included questions about labor participation and individual wages, firstly when the person started HAART after haven received the HIV diagnosis and secondarily, at the time of answering the questionnaire. This approach is called the human capital approach which estimates productivity losses through changes in labor status and in wages [12, 26].

### Other costs

The cost of transportation which is the main cost variable here was also obtained using the same questionnaire that was used to obtain the opportunity cost. The unit costs were based on fares announced by the Ghana Private Road and Transport Union as at October 2009. The transportation cost took into consideration the period of counseling that also involved an adherence monitor.

### Total costs

The total costs to the patients were finally estimated by the sum of the direct costs, indirect costs and other costs (transportation).

### Data analysis

Data were doubled entered into an Access database (Microsoft 2003), then imported and analyzed using EpiInfo version 3.4.3 November 8 2007. The median direct, indirect and total costs were then determined with inflation accounted for using the October 2009 US dollar rate.

### Ethical considerations

Permission was obtained from the management of Kintampo Municipal Hospital and the Association of people living with HIV/AIDS in both Kintampo North and South districts.

## Results

A total of 80 questionnaires were administered during the period of the study to clients on HAART attending the antiretroviral clinic over the period.

### Demographic information

More females than males were on HAART in this population as shown in Table 1 with the mean ages of females and males being 38.4 years and 39.4 years respectively. The proportion of clients on HAART with higher education was generally low (3.95%) as compared to those with low or no education at all (Table 2). A lot more clients 31(38.8%) still remained married than those divorced 21 (26.3%) after being diagnosed and put on HAART. However only 7 (8.8%) had lost their partners with the rest 21(26.3%) not married. Majority (96.2%) of this population was insured by the national health insurance scheme with the remaining 3.8% having to take care of other medical bills outside the HAART program.

**Table 1:** Sex distribution of clients

Sex	n	Percentage
**Male**	17	21.79
**Female**	61	78.21
**Total**	**78**	**100**

2 questionnaires excluded due to incomplete information

**Table 2:** Educational Background of clients

Educational level	n	Percentage
**Primary**	27	35.53
**Secondary**	12	15.79
**Tertiary**	3	3.95
**None**	34	44.74
**Total**	**76**	**100**

4 questionnaires excluded due to incomplete information

### Economic activity and cost

Close to 93% of the clients did not have formal employment but majority of them where however engaged in subsistence farming and petty trading (Table 3). None of the clients had their jobs affected after their diagnoses were disclosed to them. The median cost of transportation was estimated to be $US15.40 (22.00 Ghana cedis). The median total, indirect and direct annual costs to rural ambulating HIV/AIDS patients on HAART were estimated to be $US71.18 (115.16 Ghana cedis), $US2.740 (3.92 Ghana cedis) and $US53.04 (75.00 Ghana cedis) respectively.

**Table 3:** Employment Status of clients

Employment	n	Percentage
**Formal employed**	6	7.50
**Unemployed**	74	92.50
**Total**	**80**	**100**

Majority of the unemployed clients are actually engaged in subsistence farming and petty trading.

58.75% (47) of the respondents were able to disclose or give an estimate of their annual incomes resulting in a median annual earned income of $US50.21 (71.00 Ghana cedis) of which that of males was double ($US84.87) that of females ($US42.43). Meanwhile only 33.75% (27) of the respondents were able to disclose or give an estimate of their annual household incomes resulting in an estimated median annual household income of $US00.00.

## Discussion

The number of females being more than three times that of males in this study in spite of the convenient sampling method used is reflective of the sex distribution of this population since females are about three or more times that of males. From the results obtained above, it seems very clear that rural ambulating HIV/AIDS patients on HAART will have to look for extra money outside their annual income attending the antiretroviral clinic alone since they spend an extra 38% more than their median annual income. This undoubtedly has a very telling effect on their individual income in particular and household income in general. Patients could even spent more depending on how far they had to travel to get to the antiretroviral centre. This finding was not different from what was reported in the news letter of the Educational Research Network for West and Central Africa on Ghana [22]. This finding is also consistent with an out-of-pocket costs of AIDS care study done in China [13]. The high cost of transportation which forms about 30% of median annual earned income obviously has the potential of resulting in high default rate and hence high mortality rates which is consistent with what was reported by the UN Integrated Regional Information Networks [23]. The low indirect cost of $US2.74 which constitutes just a little over 5% of their annual earned income is due mainly to the fact that very few of the respondents are employed and actually have no earned income. However the very low unemployment rate in this population is an actual reflection of the entire population of the study area. This low indirect cost is however inconsistent with findings in the Canary Islands of Spain which showed a high indirect cost [12]. Similarly contrary to the findings in this study, a high indirect cost was observed in a pilot study carried out in Madrid to determine the indirect costs in HIV/AIDS ambulatory patients in Spain [20]. This study estimated both direct and indirect costs as well as other costs such as transportation cost unlike the other few studies that looked at patient level data in sub-Saharan Africa were they looked at mainly direct cost [7, 14].

The results of this study cannot be confidently generalized due to the methodological approach (convenient sampling) and also the fact that it was carried out in just one clinic for a short period of time and involving a small sample. The use of prevailing market prices as unit costs which are themselves unreliable due to fluctuating market prices somehow has an effect on the indirect cost.

This notwithstanding, the study has not fallen short of providing a more comprehensive patient level data in rural areas of Africa of cost estimates considering the weakness of the other studies [7–9, 14]. A larger study using a random sampling technique, involving more ART clinics and covering the whole year will give a better estimate of cost since the cost of transportation tends to have a seasonal variation due to in assessable roads.

## Conclusion

The findings of this pilot study give a fair idea of the costs per annum to rural ambulating HIV/AIDS patients on HAART attending antiretroviral clinics rural Ghana. Although the cost of antiretroviral drugs has been subsidized by government from $360 to $41.38 per annum, HIV/AIDS patients on HAART spend double of this subsidized amount out of their pocket seeking health care which has serious consequences especially regarding default rate. We recommend that agencies associated with HIV/AIDS activities, supplements government's effort by helping to get antiretroviral closer to the door step of patients so as to reduce this huge financial burden which constitutes more than 100% of their median annual earned income.
